# Polymer therapeutics in surgery: the next frontier

**DOI:** 10.1002/jin2.6

**Published:** 2016-04-18

**Authors:** Ernest A. Azzopardi, R. Steven Conlan, Iain S. Whitaker

**Affiliations:** ^1^ Reconstructive Surgery and Regenerative Medicine Research Unit, Institute for Life ScienceSwansea University Medical School, Swansea UniversitySingleton Park CampusSwanseaSA2 8PPUK; ^2^ The Welsh Centre for Burns and Plastic SurgeryMoriston Hospital SwanseaSwanseaSA6 6NLUK; ^3^ Institute for Life Science and Centre for NanoHealthSwansea University Medical School, Swansea UniversitySingleton Park CampusSwanseaSA2 8PPUK

**Keywords:** nanomedicine, nanotechnology, polymer

## Abstract

Polymer therapeutics is a successful branch of nanomedicine, which is now established in several facets of everyday practice. However, to our knowledge, no literature regarding the application of the underpinning principles, general safety, and potential of this versatile class to the perioperative patient has been published. This study provides an overview of polymer therapeutics applied to clinical surgery, including the evolution of this demand‐oriented scientific field, cutting‐edge concepts, its implications, and limitations, illustrated by products already in clinical use and promising ones in development. In particular, the effect of design of polymer therapeutics on biophysical and biochemical properties, the potential for targeted delivery, smart release, and safety are addressed. Emphasis is made on principles, giving examples in salient areas of demand in current surgical practice. Exposure of the practising surgeon to this versatile class is crucial to evaluate and maximise the benefits that this established field presents and to attract a new generation of clinician–scientists with the necessary knowledge mix to drive highly successful innovation.

## Background

Polymer therapeutics represents a highly successful nanomedicine class that has enjoyed extensive success from aesthetic surgery to neoadjuvant oncology, features in the top 10 US pharmaceutical sales lists, is arguably the most successful first‐generation nanomedicine class, and is well established in perioperative use (Duncan, 2014). Polymer therapeutics offer substantially different properties to conventional counterparts including passive accumulation at target sites and bioresponsive activation and lend themselves extensively to custom‐engineered solutions for specific clinical demands. A working knowledge of this burgeoning scientific field is imperative, for the surgeon to evaluate the significantly different properties compared with conventional therapy, safeguard perioperative patient safety, and contribute to the development process (Duncan and Gaspar, 2011; Gaspar and Duncan, 2009).

This study provides an overview of polymer therapeutics applied to clinical surgery in its several subspecialties, including the evolution of this demand‐oriented scientific field, cutting‐edge concepts, its implications, and drawbacks, illustrated by an extensive review of products already in clinical use and promising ones in advanced stages of development.

## Method

A comprehensive database search of MEDLINE, EMBASE, and Pubmed Central was performed. The search was limited to studies in English, using the Boolean search string “polymer and therapeutic” and “surgery” (all subspecialties). Seminal works underpinning the principal tenets of polymer therapeutics were included. Title and abstract of the primary literature were reviewed for relevance and included in the review. This literature was back and forward referenced using the Web of Knowledge™ database.

## Literature Review

### History and working definitions

Evolution towards the microscale has represented a paradigm shift in surgical practice since Carrel's studies on vessel anastomosis (Carrel, 1902). Jacobson and Suarez introduced the benefits of the operating microscope in the 1960s, followed by the first successful free muscle transplants in the 1970s and super microsurgery (Kriss and Kriss, 1998; Tamai, Komatsu, Sakamoto, et al., 1970). The adoption of these techniques across surgical specialties within coronary vessel repair, otolaryngology, and head and neck surgery bears witness to their success. The continuous surgical drive towards the infinitesimal and its attendant benefits necessarily demanded an exploration of the next frontier represented by the nanometre scale.

**Figure 1:**
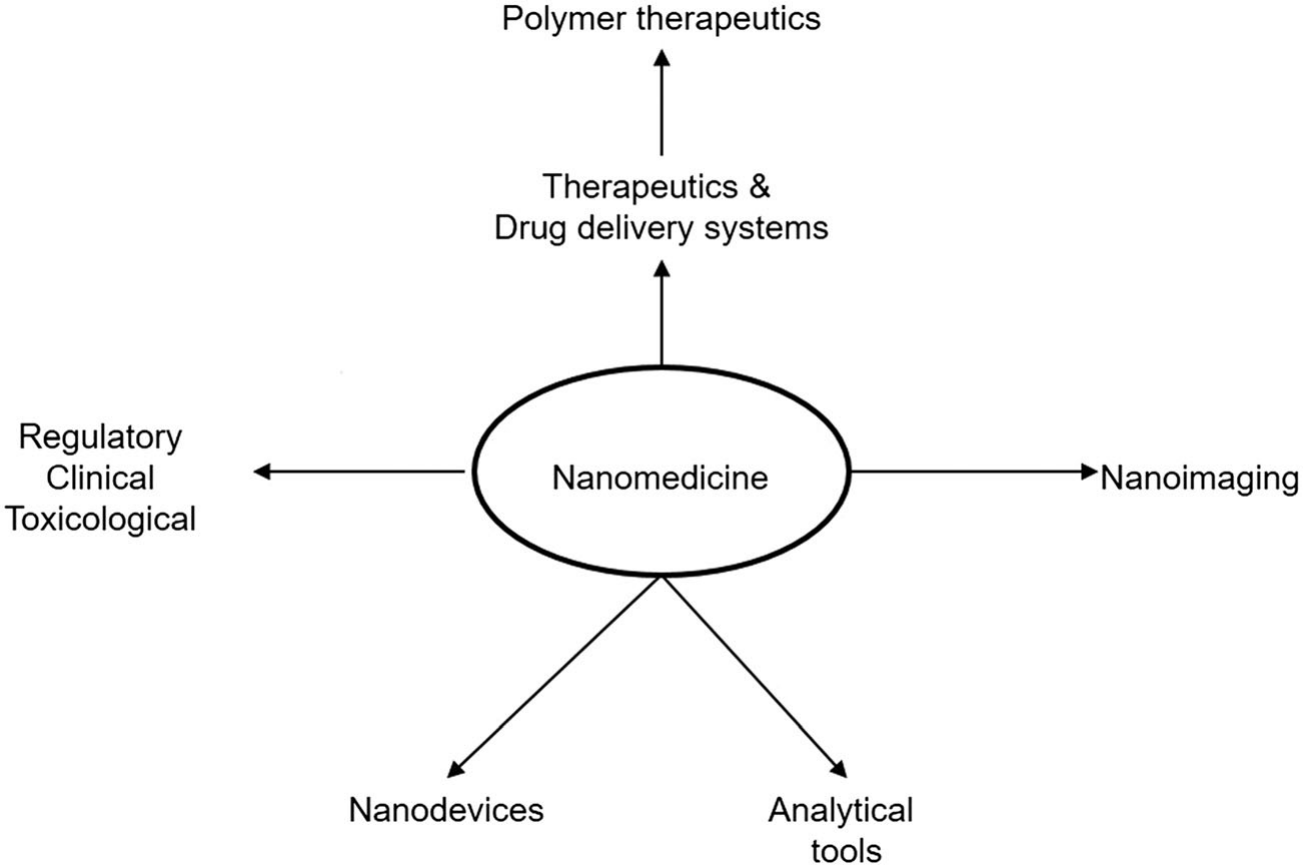
The five main subdisciplines of nanomedicine (European forward look consensus conference, 2004, and the relationship of polymer therapeutics to these subdisciplines).

**Figure 2:**
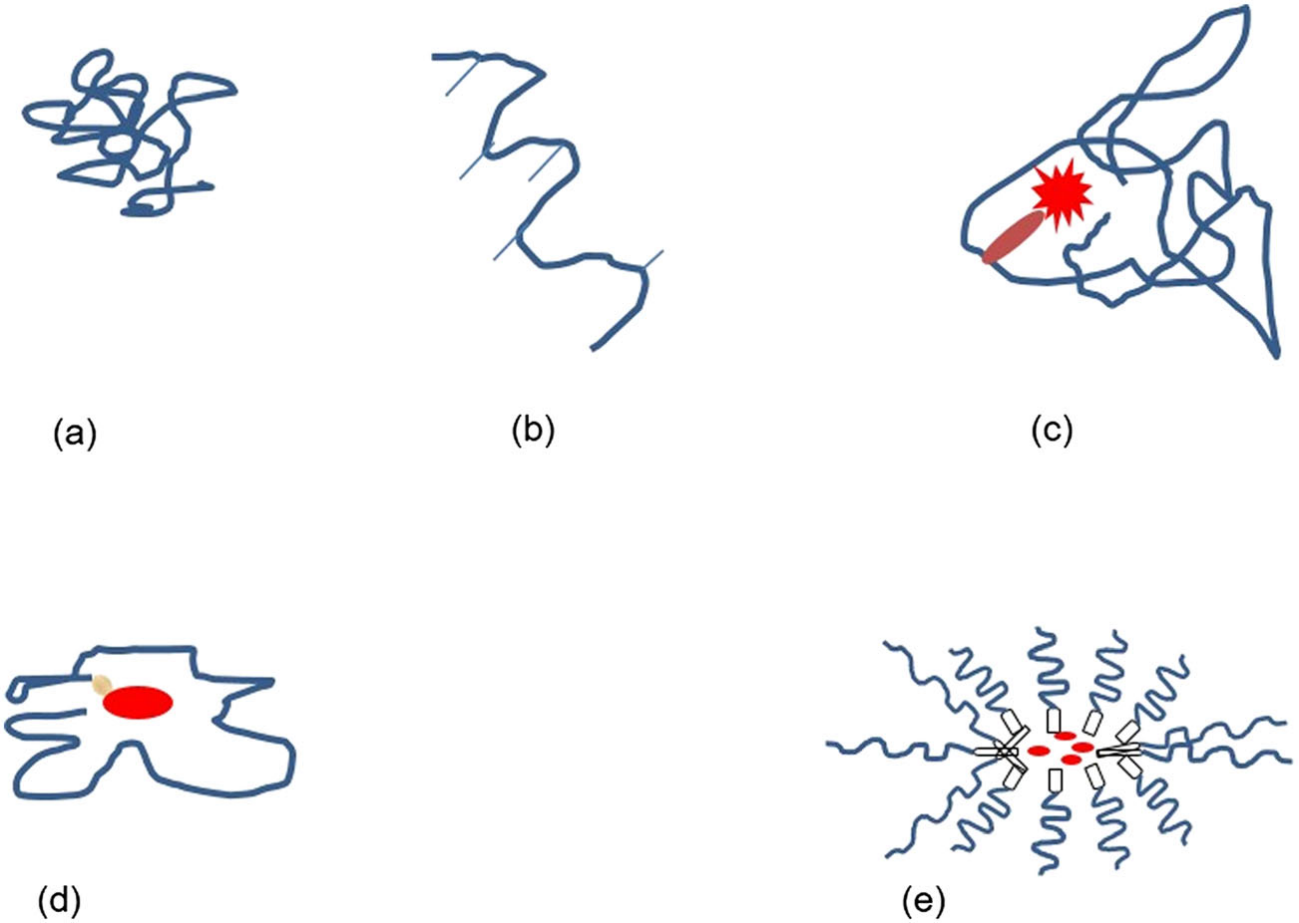
Diagrammatic representation of different types of polymer therapeutics. The presence of a water‐soluble polymer is the common denominator. Examples are given in parentheses. (a) Polymeric drug; (b) polymeric drug modified by the addition of pendant groups; (c) polymer–protein conjugate; (d) polymer–drug conjugate; and (e) PEGylated micelles.

Nanomedicine uses nanosized (between 1 and 100 nm) tools addressed toward the diagnosis, prevention, and treatment of disease – molecules that are of much larger size than conventional drugs (European Science Foundation, 2005). Modern awareness of the benefits of nanomedicine dates back to Paul Ehrlich's first low‐molecular‐weight synthetic chemical entities (Duncan, 2014). Since then, nanomedicine has expanded into five overlapping subdisciplines (Fig. 1). Polymer therapeutics are nanoscale therapeutics and drug delivery systems, pioneered by Herman Staudinger's ground‐breaking work on covalently linked macromolecules, popularised and expanded through the work of Kopeck, Ringsdorf, and Duncan (Duncan and Gaspar, 2011; Duncan and Vicent, 2013). The term “polymer therapeutics” has evolved to an umbrella term encompassing a number of heterogeneous entities including polymeric drugs, polymer–drug conjugates, and polymer–protein conjugates (Fig. 2) (Duncan, 2003). Polymer therapeutics lends itself particularly well to the concept of demand‐to‐supply research and therefore presents an important opportunity for the surgeon–scientist to translate a clinical demand to a custom‐engineered product. This demands a working knowledge of the major differences and designs distinguishing a polymer therapeutic from a conventional alternative.

### Design: custom engineering from bench to bedside and back

The common denominator for all polymer therapeutics is the possession of a water‐soluble polymer. Polymers consist of repeating component units (monomers) to produce a macromolecular structure with unique physicochemical characteristics. Several types of polymers are employed, and a simple working classification is provided in Table 1. Polymeric drugs represent the simplest form of polymer therapeutic (Fig. 2a). Here, the polymer itself is the active drug. Applications include structural tissue support, increasing lubrication, structural volume expansion such as hyaluronic acid (HA), and plasma volume expansion such as dextran.

**Table 1:** Classification of polymers and prominent examples in clinical use.

Classification	Subclasses	Polymer example	Clinical example	Surgical specialty
Biodegradability	Nonbiodegradable	PEG	PEG recombinant	Gastrointestinal
			IFN α2b (hepatitis C)	Hepatobiliary
			PEG‐IFN_α‐2b_	Melanoma surgery
	Biodegradable	HA	Dermal fillers (aesthetic surgery)	Aesthetic (Bray et al., 2010; Madani et al., 2011; Pavelka and Uebelhart, 2011)
				Orthopaedic (Duncan and Vicent, 2013)
Monomer	Homopolymer	PEG	PEG–doxorubicin (ovarian cancer)	Gynaecology
				Pelvic
			PEG‐Erythropoietin	Anaemia (Duncan and Vicent, 2013)
			PEG‐anti‐TNF Fab	Rheumatoid arthritis
	Copolymer	Random amino acid copolymer*	Glatiramer acetate (multiple sclerosis)	Neurosurgery
		Polyvinylpyrrolidone	Povidone iodide	Antiseptic (Ascher, Bayerl, Brun, et al., 2011)
				Ubiquitous
				Dressings
				Hand scrub
Shape (examples)	Linear	Dextrin	Dextrin	Nephrology
	Branched	PEG (branched)	PEG‐IFN α2a (hepatitis C)	Gastrointestinal
				Hepatobiliary
	Dendrimeric	Lysine‐based dendrimer 7013	3% Carbopol formulation (intravaginal viricide versus HIV)	Obstetrics
				Gynaecology

PEG, poly(ethyl glycol); HA, hyaluronic acid; IFN α2a, interferon alpha 2a; IFN α2b, interferon alpha 2b.

* Glutamic acid, lysine, alanine, and tyrosine.

The polymer may be customised to a clinical niche by the induction of pendant branches along the main backbone chain. In the natural form, injectable HA is locally degraded within 48 h (Fakhari and Berkland, 2013). However, HA cross‐linking increases resistance to biodegradability by endogenous human hyaluronidase enzyme (further applications of HA are considered in Macromolecular Status and the Enhanced Permeability Retention Effect section). Conjugation to 1,4‐butanediol diglycidal ether and divinyl sulphone results in very predictable degradation over time. 1,4‐Butanediol diglycidal ether and divinyl sulphone are currently the only two cross‐linking agents licenced for dermal injectable use (Bray, Hopkins, and Roberts, 2010; Fullana and Wnek, 2013). Dextrin is another example of a Food and Drug Administration (FDA)‐approved, largely linear polymer, which is rapidly digested by naturally occurring amylase at physiological concentrations. However, it can be modified by increasing amounts of succinoylation (Fig. 2b). In the presence of physiological amylase activity, this provides a highly predictable and custom‐engineered degradation rates and differentially, in microenvironments within the body, where amylase might accumulate (Azzopardi, Camilleri, Moseley, et al., 2013a).

“Pendant groups” can additionally serve as “linkers” enabling chemical bonding of a polymer to a bioactive molecule of interest, a process termed conjugation. For example, HA has been conjugated to various molecules of interest including antiinterleukin‐1β and antitumour necrosis growth factor‐α monoclonal antibodies (Duncan and Vicent, 2013). Polymer–drug conjugates are arguably the commonest forms of polymer therapeutics and, like other polymer therapeutic subclasses, have properties substantially distinct from the conventional alternatives (Table 2). Additionally, conjugation creates new, original macromolecules, affording intellectual property space, which is crucial for successful commercialisation (Pinter, Horvath, Bujdoso, et al., 2009).

**Table 2:** Examples of properties conferred by conjugation in polymer therapeutics.

Polymer therapeutic	Conventional alternative
Improved biological efficacy (Duncan, 2003)	Conventional biologic efficacy
Extended plasma circulation time (Koburger, Hübner, Braun, et al., 2010)	Conventional plasma residence times, clearance, and degradation
Shielding from immunogenicity and premature biofouling and clearance (Werle and Bernkop‐Schnürch, 2006)	Conventional risk of immunological reaction, sequestration, and clearance
Enhanced permeability and retention effect (macromolecule) (Maeda, 2010; Maeda, Bharate, and Daruwalla, 2009)	Indiscriminate distribution (conventional small molecule)
Potential for “masking/unmasking” and locally triggered reinstatement of bioactivity (biodegradable polymers)	N/A

### Macromolecular status and the enhanced permeability retention effect

Conjugating a conventional “small molecule” to a polymer of adequate size creates a macromolecule. As molecular size increases, filtration at the glomerulus decreases, a happy state of affairs if the newly synthesised molecule is to avoid perioperative nephrotoxicity whilst simultaneously increasing plasma residence time (Azzopardi, Ferguson, and Thomas, 2014a). Colistin, for example, is increasingly used to treat multidrug‐resistant surgical infection. Its use is limited by its reputation for nephrotoxicity (Azzopardi, Boyce, Thomas, et al., 2013b). However, conjugation to dextrin, using succinoyl linking groups, resulted in substantial reduction of kidney clearance and absence of any observed clinical toxicity in vivo within Sprague Dawley rats (Azzopardi et al., 2014a).

Conversion of a conventional “small molecule” antibiotic into a macromolecule may benefit from passive, size‐based targeting to an inflamed area (Azzopardi, Ferguson, and Thomas, 2013c). The enhanced permeability and retention (EPR) effect refers to the ability of macromolecules to passively accumulate at the site of enhanced vascular permeability and be selectively retained therein (Maeda, 2012). This principle has been extensively applied to the design of clinically successful antineoplastic and neoadjuvant drugs, inflammatory diseases such as rheumatoid arthritis, and other chronic conditions (Duncan, 2011; Duncan and Gaspar, 2011; Duncan and Vicent, 2013; Hardwicke, Hart, Bell, et al., 2011). This concept has been extensively exploited in the design of next generation of chemotherapeutic agents entering clinical practice, such as polyethyleneglycol (PEG)‐asparaginase (Oncaspar^R^, PEG‐L‐asparaginase, Sigma‐Tau Pharmaceuticals, Inc., Gaithersburg, MD, USA) (Duncan, 2011).

It is worth noting that whilst EPR has been demonstrated to be highly successful in preclinical species, it has been less successful clinically. This could be accounted for by several possibilities including failure of the construct to address a specific niche, including real‐life drug interactions that might diminish the efficacy of EPR and does highlight the need for closer clinician collaboration in husbanding the strategy for construction of a particular polymer therapeutic to address a particular clinical niche. Meanwhile, targeting that is augmented by either specific ligands or radiotherapy may be more prerequisite for treatment of diseases that are otherwise not terminal.

### Bioresponsive polymer therapeutics and smart release

Classically, nonbiodegradable synthetic polymers, including PEG, *N*‐(2‐hydroxypropyl) methacrylamide (HPMA), and poly(lactic‐co‐glycolic) acid, comprise the majority of clinical success stories (Pasut and Veronese, 2009; Duncan, 2009). PEG conjugates are clinically well tolerated and extensively used. The surgical community is well versed with applications of some of these polymers in suture materials (Najibi, Banglmeier, Matta, et al., 2010). More recently, however, the advantages of biodegradable polymers, including the ability to respond to biological stimuli, have been intensively studied.

**Figure 3:**
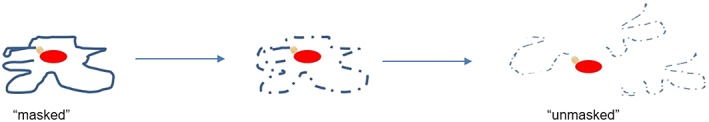
The polymer mask–unmask protein therapy principle. During transit, the polymer “masks” the bioactive from the body, at the same time shielding the body from potential toxicity. At the target site, the bioresponsive polymer is degraded (using various approaches) to release back the bioactive molecule, with its activity reinstated.

The degradation of bioresponsive polymers can be custom engineered to suit particular clinical demands (Design: Custom Engineering from Bench to Bedside and Back section), based on either exogenous enzymes co‐administered to the patient (in their native form, or even themselves as polymer–enzyme conjugates) (Duncan, Gac‐Breton, Keane, et al., 2001). More recently, an elegant approach for “shielding” the bioactive payload in transit, followed by localised enzymatic controlled release and restitution of bioactivity, has been described (Duncan, Gilbert, Carbajo, et al., 2008). This depends on conjugation to biodegradable polymers. The polymer masking–unmasking protein therapy principle involves a multifunctional biodegradable polymer to envelope the payload of interest whilst allowing locally triggered polymer degradation and reinstatement of the masked bioactive's activity (Fig. 3) (Duncan et al., 2008). The masked conjugate offers improved biological efficacy, extended plasma circulation time, and reduced proteolytic degradation and protein immunogenicity (Roberts, Bentley, and Harris, 2002; Werle and Bernkop‐Schnürch, 2006). Locally triggered unmasking at the intended site allows controlled reinstatement of bioactivity. Triggered release can be effectively controlled and predicted by the length of the polymer and the amount of linker modification (Azzopardi, 2013b).

### Physicochemical customisation

Conjugation can also radically alter mechanical, tensile, and viscoelastic properties. The effect of HA conjugation on its stability and potential as controlled release mechanism has already been referred to in Design: Custom Engineering from Bench to Bedside and Back section. Additionally, HA enjoys a central role in reconstructive surgery and regenerative medicine. It is present in most body fluids and tissues, including dermis, vitreous humour, and hyaline cartilage (Azzopardi, Ferguson, and Thomas, 2013d; Fakhari and Berkland, 2013; Griffith, 2000; Zheng Shu, Liu, Palumbo, et al., 2004). Its avidity to water molecules provides viscoelastic properties, but HA also simultaneously acts as mechanical support, biological scaffold, making it a prime target for cosmetic and facial rejuvenation applications (Garg and Hales, 2004). At a cellular level, its roles are multiple and complex. It is binding other matrix molecules, guiding cell proliferation and differentiation, and guides morphogenesis, wound repair, and inflammation (Zheng Shu et al., 2004). Recent studies on naked mole rats report that HA plays an important role in mediating its remarkable resistance to cancer (Tian, Azpurua, Hine, et al., 2013). It is therefore discussed as an elegant example of the versatile potential for polymer therapeutics to custom‐engineered solutions to complex clinical problems.

Photocross‐linked HA affords mechanical stability and has found favour in cartilage tissue engineering, cardiac repair, molecule delivery, valvular engineering, control of stem cell behaviour, and microdevices (Burdick and Prestwich, 2011). Chondrocytes within these modified HA hydrogels also resulted in cartilage production within the porous network (Allison and Grande‐Allen, 2006). Use of HA for nonsurgical facial rejuvenation is well established (Greco, Antunes, and Yellin, 2012).

## In the Pipeline

There are a number of promising polymer therapeutics in advanced stages of experimentation in vivo relevant to the practising surgeon, and salient examples are summarised in Table 3. Examples serve to expose the reader to the opportunities presented by this field in several aspects for future surgical practice.

**Table 3:** Examples of promising polymer therapeutics in development.

Polymer	Polymer therapeutic	Examples and development
Dextran	^99^Tc tilmanocept	Identification of sentinel lymph nodes in breast cancer and melanoma (phase III)
HPMA	HPMA–copolymer–diaminocyclohexyl (DACH) platinate	Malignant melanoma in phase 2 clinical trial (Maeda, Fang, Inutsuka, et al., 2003)
Succinoylated dextrin	Succinoylated dextrin–colistin	In vivo preclinical phase (Azzopardi et al., 2011a)
	Succinoylated dextrin‐recombinant EGF	In vivo preclinical phase (Duncan and Vicent, 2010; Hardwicke et al., 2010, 2011; Serbest et al., 2005)
	Succinoylated dextrin‐phospholipase‐A2	In vitro breast cancer (Hardwicke, Ferguson, Moseley, et al., 2008b)
Poloxamer	Various	In vivo preclinical phase (Medina et al., 2011; Sikkink et al., 2009)

HA, hyaluronic acid; HPMA, *N*‐(2‐hydroxypropyl) methacrylamide.

### Pharmacosurgery: the potential of modality combination treatment

Polymer therapeutics offers the exciting ability to custom‐designed molecules for specific perioperative demands. Such pharmacosurgical therapy may have the potential to increase the scope and magnitude of therapy beyond the conventional. The following section lists some common examples of polymer therapeutics in surgical practice as well, high‐profile candidates for clinical entry, and specific areas of development including management of surgical site infection, oncological surgery, and radiotherapy.

Localised lymphatic distortion frequently accompanies surgically treated conditions in oncological surgery, such as lymphatic dissection. It is rational to entertain the notion that this significant lymphatic distortion may serve to amplify and prolong an EPR effect, making it possible to specifically engineer bioactive entities to selectively target the affected region. Moreover, the EPR (Maeda, 2012) effect is enhanced by radiotherapy, and targeting of polymer–drug conjugates by radiotherapy has been clinically observed (Ke, Milas, Charnsangavej, et al., 2001). These examples postulate the emergence of “Pharmacosurgery” as the synergistic perioperative combination of surgery and bioactive pharmacological agents, more efficacious in simultaneous administration than the individual therapeutic modality, as an avenue of significant interest in perioperative diagnosis and treatment.

### Targeted antibiotic delivery to surgical site infections

Several macromolecular entities have been reported to accumulate at the infected site, despite the absence of specific receptors or transport mechanisms (Evans, Evans, and Gorbach, 1973; Laverman, Boerman, Oyen, et al., 1999, 2001; Laverman, Dams, Storm, et al., 2001b; Melendez‐Alafort, Nadali, Pasut, et al., 2009). Several studies report that the notion of rapid, passive, size‐based accumulation around foci of acute infection is feasible (Azzopardi 2013a). It presents the ability to target a dose of antibiotic selectively and rapidly towards surgical site infection. Such examples include PEG‐ubiquicidin, ^99^Tc‐labelled poly(ethylene glycol)‐coated liposomes (^99m^Tc‐PEG‐liposomes), and gallium‐transferrin (Dams, Reijnen, Oyen, et al., 1999; Laverman et al., 1999, 2001; Oyen, Boerman, Storm, et al., 1996). ^99^Tc‐labelled proteins including aprotinin (6512 g/mol) allow rapid localisation around infected foci induced in vivo animal models (2 h), and their concentration was up to 6.5 times higher than control tissue (Komarek, Kleisner, Komarkova, et al., 2005). A correlation between intraabdominal abscesses and the magnitude of effect of EPR has also been reported with some compounds (Sikkink, Reijnen, Laverman, et al., 2009). Polymer therapeutics therefore presents the potential for a clinically feasible avenue to the management of multidrug‐resistant surgical site infection.

### Polymer therapeutics in tissue regeneration

Poloxamers are nonionic triblock copolymers composed of a central hydrophobic chain of polyoxypropylene (poly(propylene oxide)) flanked by two hydrophilic chains of polyoxyethylene (poly(ethylene oxide)). It has been recently shown that improvement in apoptosis and cell viability mediated by poloxamer 188 may lead to increased fat graft viability (Medina, Nguyen, Kirkham, et al., 2011) and recovery of neuronal tissue from mechanical injury (Serbest, Horwitz, and Barbee, 2005).

Recently, succinoylated dextrin conjugated to recombinant EGF has shown promise as a controlled release approach with excellent results on in vivo animal models of chronic wounds (Hardwicke, Moseley, Stephens, et al., 2010; Hardwicke, Schmaljohann, Boyce, et al., 2008a; Hardwicke et al., 2011). This product combines the aim of protecting the growth factor from the harsh chronic wound fluid environment but allows reinstatement of its activity when exposed to α‐amylase using the polymer masking–unmasking protein therapy concept.

### Polymer therapeutics in oncological surgery

Despite its widespread acceptance, literature reports that injection of blue dye at the site of primary tumour for the identification of dye sentinel lymph node biopsy remains largely nonstandardised (Sondak, King, Zager, et al., 2013). Isosulfan blue or Patent Blue V dye followed by methylene blue dye has been used for sentinel lymph node mapping, despite controversy about their comparability and safety (Liu, Truini, and Ariyan, 2008; Neves, Reynolds, Hazard, et al., 2011). The combined use of radiolabelled colloid is also widespread, and recently, the FDA approved ^99^Technetium–sulphur colloid for sentinel lymph node identification in breast cancer (Pharmalucence press release, n.d.). However, this approval was based on retrospective data showing the noninferiority of the latter to the blue dye method. Tilmanocept is a recently described mannosylated dextran‐based polymer therapeutic for sentinel lymph node imaging, which may offer an innovative solution for melanoma and breast cancer patients and requires no manipulation before injection. It is reported to bind tightly to CD 206 mannose receptors on the surface of reticuloendothelial cells resident in lymph nodes for up to 30 h (Sondak et al., 2013).

More recently, superparamagnetic iron oxide contrast agent injected subcutaneously into the breast rather than intravenously has gained FDA approval for the purpose of sentinel lymph node identification and has demonstrated an identification rate in humans that is noninferior to the standard technique (Duncan and Gaspar, 2011).

The EPR effect has been widely adopted to target biologically active payloads to solid tumours. In 2011, PEG‐interferon α‐2b has recently been approved as an adjuvant therapy for the treatment of high‐risk melanoma. An open‐label randomised study of resectable stage III melanoma reported a significantly increased median recurrence‐free survival. However, overall survival was not significantly different to controls (Ditrolio, Simeone, DI Lorenzo, et al., 2012). HPMA–copolymer–DACH platinate has recently entered phase II trials for melanoma. Similarly, first clinical studies with transferrin‐targeted polymer–cyclodextrin nanoparticle small interfering ribonucleic acid delivery systems have demonstrated nanoparticle localisation to melanoma tissue (Davis, 2009; Galanski and Keppler, 2012).

## Safety of Polymer Therapeutics

The clinical success of several polymers as plasma expanders, and egregious fall from grace of others, serves to illustrate the imperative for clinical knowledge of this field in the interest of patient safety. This section summarises current controversies and cautions towards polymer therapeutics in surgical practice (Table 4).

**Table 4:** Examples of salient safety issues with particular polymers.

Polymer	Polymer therapeutic
Nondegradable polymers	Theoretical risk of toxic accumulation in lysosomal storage‐like disorders
Dextrans	May generate an immunoglobulin‐M response (Azzopardi, Ferguson, and Thomas, 2011b)
	Degraded slowly (Azzopardi et al., 2011b; Battisto and Pappas, 1973)
	Tend to form nondegradable products during chemical modification (Battisto and Pappas, 1973)
HES	HES fractions may cause hypersensitivity and interfere with coagulation processes causing haemorrhage (Vercauteren, Bruneel, Schacht, et al., 1990; Zarychanski et al., 2013)
Metabolites	As with all drugs, it should be ensured that the metabolites are assessed for any adverse/toxicological reaction (Duncan and Vicent, 2013; Gaspar and Duncan, 2009)

HES, hydroxyethyl starch.

Hydroxyethyl starch was used until recently to expand the volume of circulating plasma but carried an increased risk of renal dysfunction and mortality over a 90‐day follow‐up in patients who received hydroxyethyl starch compared with crystalloids. Increased mortality in patients with sepsis was also observed prompting their UK‐wide recall (Brunkhorst, Engel, Bloos, et al., 2008; MHRA, 2013; Myburgh, Finfer, Bellomo, et al., 2012; Perner, Haase, Guttormsen, et al., 2012; Zarychanski, Abou‐Setta, Turgeon, et al., 2013). Clinical success of dextran has been mixed. Its use as volume expander has been limited by a reported aptitude for causing renal impairment (Bhatt, Neppalli, Kelley, et al., 2011). Its anticoagulant properties have, however, found clinical purpose in some quarters in microvascular surgery, although this is presently contended (Djohan, Gage, and Bernard, 2010; Riva, Chen, Tan, et al., 2012) rather than the limited quantities associated with administration of medicinals (Azzopardi, McWilliams, Iyer, et al., 2009; Boussekey, Darmon, Langlois, et al., 2010; Gattas, Dan, Myburgh, et al., 2012). It is likely that these effects are clinically relevant with the significant amounts used as volume expanders rather than the limited quantities that would be used in their role as polymers for drug conjugation. However, these cautionary tales underscore the importance of the surgeon's familiarity with potential polymer therapeutic side, as the ultimate custodian of patients' safety.

Finally, renal failure is a frequent perioperative complication (Mitchell, 2013). An advantage of macromolecular constructs is increased plasma residence time by way of avoiding filtration at the kidney, thereby decreasing unwanted nephrotoxicity. The use of biodegradable polymers like dextrin and HA present the potential for degradation and metabolism into normally produced metabolites such as glucose, maltose, and isomaltose (dextrin, dextran, and starch) or amino acids (HA). However, nonbiodegradable polymers such as PEG do present the theoretical risk of toxic accumulation lysosomal storage‐like diseases, and other metabolic aberrations become theoretically possible, especially when chronic administration would be anticipated (Gaspar and Duncan, 2009).

The US Nanotechnology Characterisation Laboratory's work is of interest to this section in having defined an assay cascade with which to assess, in a timely and rationalised manner, the physical attributes, in vitro biological properties, and in vivo compatibility (in animal models) of submitted requests, through which it standardises the preclinical characterisation of nanomaterials intended for cancer therapeutics and diagnostics (Gaspar and Duncan, 2009). To the same end, a sister European institution, the European counterpart facility, EU‐Nanotechnology Characterisation Laboratory, has been recently launched (Carrel, 1902).

## Discussion

Polymer therapeutics has evolved into a clinically successful branch of nanomedicine. The extensive versatility of the building blocks themselves (polymer, linker, and drug) and the custom‐engineering strategies available open up exciting innovative and sustainable horizons to pressing surgical problems such as surgical site infection, tissue regeneration, reconstruction, and oncology.

However, transferring the benefits to the bedside requires dual expertise in surgery and polymer therapeutics. Central to the success of polymer therapeutics, therefore, is that the demand‐to‐supply ethos of the field is nourished by the dual training of surgeon–scientists. Clinical academics are ideally placed between demand and supply ends of the research translational chain, and it is essential that a new generation of clinician scientists is attracted to the field if the success stories of polymer therapeutics in other clinical areas are to be replicated across the surgical specialties. The surgical community does not afford to be left out from being intimately involved in the development of this technology and underlying paradigms, and this is being pioneered by clinical–academic programmes in the UK to some extent, and nascent training programmes geared toward the dual clinical–academic development have yielded interesting results (Azzopardi, Ferguson, and Thomas, 2011a, 2013c, 2014b; Azzopardi et al., 2013, 2013a; Madani, Naderi, Dissanayake, et al., 2011). More importantly, exposure to this ongoing revolution may help maintain and develop a clinically oriented, demand‐driven ethos in this specialism. It is essential to attract a new generation of clinician–scientists with the necessary knowledge mix to drive highly successful translational innovation whilst preserving the surgeon's role as the ultimate guardian of patient safety.

This study has provided an overview of polymer therapeutics applied to clinical surgery, including the evolution of this demand‐oriented scientific field, cutting‐edge concepts, its implications, and drawbacks, illustrated by an overview of products already in clinical use and promising ones in advanced stages of development. This journal encourages and welcomes manuscripts situated at the interface between the disciplines of clinical surgery regenerative medicine, cell biology, and pharmaceutical sciences. It is time for the surgical community to step up to the plate.

## Conflict of Interest

The authors explicitly declare no conflict of interest whatsoever in this paper.
